# Low-intensity internet-delivered treatment for generalized anxiety symptoms in routine care: protocol for a randomized controlled trial

**DOI:** 10.1186/1745-6215-15-145

**Published:** 2014-04-27

**Authors:** Derek Richards, Ladislav Timulak, Gavin Doherty, John Sharry, Orla McLoughlin, Chuck Rashleigh, Amy Colla, Ciara Joyce

**Affiliations:** 1 SilverCloud Health, The Priory, John’s Street West, Dublin 8, Ireland; 2 School of Psychology, University of Dublin, Trinity College, Dublin 2, Ireland; 3 School of Computer Science & Statistics, University of Dublin, Trinity College, Dublin 2, Ireland; 4 Mater Misericordiae University Hospital, Eccles St., Dublin 7, Ireland; 5 University of Dublin, Trinity College, Dublin 2, Ireland

**Keywords:** Generalized anxiety disorder, Online interventions, Treatment, Students, CBT, Randomized trial

## Abstract

**Background:**

Worldwide prevalence of generalized anxiety disorder (GAD) is considered high; in Europe lifetime prevalence has been estimated at 4.3 to 5.9%. High levels of anxiety disorders have been reported in university students, affecting 25 to 30% of the population. Young adults are some of the most vulnerable for the onset of mental health disorders and any stressors may act as a catalyst for their onset. The absence of resources can often mean that many do not seek treatment. Other factors that impede access to resources include such things as a lack of trained professionals, personal stigma, and waiting lists. Anxiety disorders can be treated successfully; indeed brief forms of cognitive-behavior therapy have been recommended. One potential avenue for research and development is that of delivering low-intensity interventions online for students with GAD. Therefore, the current study seeks to investigate the potential effectiveness for a low-intensity online CBT-based treatment for GAD in a service-based setting; implemented as one step in a stepped-care model.

**Methods/Design:**

The research is a service-based effectiveness study utilizing a randomized waiting-list controlled design. The active intervention consists of six weekly modules of online CBT. Participants are assigned a supporter who provides weekly post-session feedback on progress and exercises. Participants will complete the GAD-7 as the primary outcome measure. Secondary outcomes include pathological worry, depression and measures of well-being. At three-months follow-up data will be collected using the GAD-7, BDI-II, PSWQ, ED-Q5 and WSAS. Post-session data will be collected on significant in-session events in treatment (HAT). A satisfaction with treatment measure will be administered post-treatment (SAT).

**Discussion:**

The study will be a contribution to the potential for a low-intensity internet-delivered program implemented in a service-based setting; implemented as one step in a stepped-care model. The study will be a contribution to the already established work in online treatments for anxiety worldwide. The study will assess the utility of an innovative digital health solution (SilverCloud) to deliver such interventions.

**Trial registration:**

Current Controlled Trials ISRCTN16303842.

## Background

Anxiety disorders rank high among the most common mental health disorders worldwide [1]. Anxiety disorders include generalized anxiety disorder (GAD), panic disorder and agoraphobia (PD/A), panic attacks (PA), specific phobia (SP), and social anxiety disorder (SAD) [2]. In general, anxiety is characterized by excessive worry about everyday events, irrespective of whether they are internal or external or originating in the past, present or future [2]. In Europe, 12-month prevalence of anxiety disorders has been estimated at 14% [3-5].

GAD is typically considered a chronic condition; a homogenous disorder characterized by excessive worry affecting several domains including restlessness, fatigue, difficulties concentrating, muscle tension, sleep disturbances, and irritability [6]. In Europe the lifetime prevalence of GAD has been estimated at 4.3 to 5.9%, with a 12-month prevalence of 1.2 to 1.9%, yet only a very small percentage seek treatment [7]. GAD, like other mental health disorders, often presents with comorbidity - principally mood disorders or other types of anxiety disorder [4,8]. GAD is associated with significant deleterious effects: economic, personal, intrapersonal and societal [9,10], which can cause significant impairment and reduced quality of life for an individual and their family [11].

### Anxiety and students

Several studies have reported elevated levels of anxiety and stress in university students [12]. The prevalence of anxiety in high school and university students has been reported to be between 25 and 30%, in response to their academic demands [13,14]. Academic stress is highly prevalent and it can contribute significantly to anxiety and depression among college students. For instance, a recent survey of young adult students in Ireland using the Depression Anxiety Stress Scale (DASS-21) [15], showed levels of clinical anxiety at 36% (8% in the mild range, 15% in the moderate range, 5% in the severe and a further 9% in the very severe range) and 30% with clinical symptoms of stress (10% in the mild range, 10% in the moderate range and 10% in the severe (7%) to very severe (3%) range) [16].

The transition to university and the management of the resulting academic demands can be a stressful time for many students. The majority of students are at a developmental stage where the onset of mental health difficulties can arise and any stressors may act as a catalyst for their onset [12,17,18]. Indeed, young adults aged between 17 and 25 are at high risk of developing a serious mental illness such as an anxiety disorder, and whilst sometimes mental disorders can be difficult to diagnose early on, the risk of delayed diagnosis is often associated with treatment resistance and poorer longer-term outcomes [12]. The result of a lack of opportunity for early diagnosis and treatment can often mean academic failure and dropping out of university. Furthermore, any such underachievement or failure can have long-term consequences on self-esteem and progress in future life [12].

Since the publication of the Royal College of Psychiatrists updated report (2011) on the mental health of students at university, there is no evidence of the abating of the concerns that were previously raised [18] he demand for counseling and mental health services among students has only increased [12]. This is due to a broad range of factors, including the rise of family breakdowns, the increase in student monetary contributions to university, the changing demographic of the student population (with a greater diversity of international students), and the case that many students now have to work to survive, places an increased demand not only on their time but also their mental health and wellbeing [12].

### Treating anxiety disorders

Anxiety can be treated successfully through disorder specific treatment plans or treatments that target common elements and symptoms across anxiety disorders [19,20]. Treatments for GAD include pharmacological and psychological and both have demonstrated their efficacy [21]. By far the most extensively researched psychological treatment for GAD is cognitive-behavior therapy (CBT) which has been shown to be a highly effective treatment for GAD [22]. Different cognitive and behavioral techniques compose any treatment for GAD. They can include cognitive restructuring, behavioral exposure, worry exposure (staying with feared outcomes), relaxation training, and problem solving, among others. CBT for GAD aims to help the user overcome emotional avoidance and learn that their anxiety is not debilitating, but can be managed and indeed recede over time [23-25].

To date a number of theoretical models have been proposed for GAD [26]. Perhaps the most well-defined and empirically supported of these are the Avoidance Model of Worry and GAD (AMW) [27] and the Intolerance of Uncertainty Model (IUM) [28]. Both have gathered supporting empirical evidence for their central constructs and have developed and researched treatment protocols based on their model [26]. The IUM is largely a cognitive model explaining the pathogenesis of GAD, whereas the AMW is an integrated model that includes cognitive alongside emotional and behavioral components. The treatment protocol used in this study is largely based on the AMW model of GAD.

It is the case that many with anxiety disorders have no diagnosis nor seek treatment [29,30]. Consequently, mental health disorders such as anxiety disorders often go undetected, especially where accessing psychological services is difficult and/or services are overburdened, as is the case for many university institutions [12,31]. Additional barriers to accessing treatment exist, such as a lack of available trained professionals, waiting lists, lack of motivation for change, negative perception of psychological treatments, indirect costs of treatment (and direct costs in some cases), personal difficulty such as stigma, and low mental health literacy. Each of this barriers can play an important role in choosing whether or not to seek diagnosis and treatment [32,33].

CBT as a brief psychological interventions has demonstrated its efficacy and has the potential to significantly reduce the burden of anxiety disorders [34]. CBT is highly suitable to being delivered as a low-intensity intervention within a model of stepped-care [35]. Ireland is far behind its European neighbors in developing and implementing stepped-care models for mental health service delivery that involve both low-intensity (such as bibliotherapy) and high-intensity (such as face-to-face therapy) interventions, despite it being recommended as best practice [36] and its demonstrated success to date [37].

### Stepped-care model of treatment

The central idea in developing stepped-care models in mental health is to extend access [38]. In recent years attempts to overcome barriers to access have been addressed through the development and implementation of a wide range of low-intensity interventions including internet-delivered treatment programs. Programs have been developed and employed in the treatment of a range of disorders and their results support their efficacy [39-42]. Internet-delivered treatments for anxiety disorders have included interventions for panic disorder, SAD, SP, and posttraumatic stress and they have established findings that support their potential effectiveness and efficacy [43-47].

More precisely, a number of studies have investigated the potential for internet-delivered treatments for GAD and they have reported significant post-treatment and follow-up gains [33,48-51] similar to those found in face-to-face treatment studies [52]. A recent meta-analysis of internet-delivered treatments for GAD demonstrated large post-treatment effects for GAD symptoms (*d* = 0.91) and pathological worry (*d* = 0.73) in favor of the active interventions when compared to waiting list control groups [53]. In addition, online studies for anxiety treatment that provide human support yield enhanced results when compared to those with no human support [54]. The demand for psychological treatments will never be met from high-intensity therapy, therefore internet-delivered treatments are a valuable alternative.

### Delivering online low-intensity interventions for GAD

The internet offers the possibility of delivering a treatment intervention at low cost and perhaps overcoming some of the barriers to access mentioned earlier. Online technologies can deliver treatment incrementally and in an engaging way. Furthermore, internet penetration in Ireland is at 76.8% [55] and it is likely that it is an attractive medium, especially for younger people as they are already high users of the internet and related tools. In a recent Irish survey that asked a student sample about the places they were likely to use as a source of support, the internet was the highest scoring answer at 78%, above friends, parents, doctors, or other professionals [16].

Some previous research has assessed the relevance of low-intensity internet-delivered treatments for anxiety disorders in service-based settings [56-58]. However, only a handful of studies have investigated the efficacy and effectiveness of low-intensity internet-delivered treatments for GAD [33,48-51]. The current study seeks to make a contribution towards understanding the relevance of a low-intensity internet-delivered treatment for GAD in a service-based setting in Ireland.

Therefore, using established CBT principles informing skills and strategies for the management of GAD in an integrated disorder-specific treatment plan, the study aims to deploy these using a novel digital health software platform (SilverCloud SilverCloudHealth Ltd., The Priory, John’s Street West, Dublin 8, Ireland.) that integrates a number of innovative engagement strategies for improving the user experience: personal, interactive, supportive, and social [59]. The details of the platform and the content of treatment are described in more detail below.

### Dropout from online treatments

A problem that has faced online treatments in general, and more particularly self-administered treatments without support, is that of dropout [60]. Dropout is a continued cause of concern as it is suggested that completing the entire course will benefit users, although several online studies have reported benefits for users who have not completed the entire course of treatment [61]. Some studies have collected information regarding dropout, suggesting difficulties using the computer, negative features of the program, perceiving the course as too demanding, poor clinical progress, receiving alternative treatment, feeling better, lack of time, and problems understanding the computer program [42,62]. The technology used in the current study has been specifically designed to include a number of engagement strategies for improving the user experience: personal, interactive, supportive, and social. An investigation of dropout will be valuable in assessing the user’s experience of online delivery.

### Objectives of the trial

The study aims to implement and evaluate the effectiveness of a low-intensity online self-administered treatment for anxiety (with support) for students with GAD symptoms. There are four research questions. Firstly, can an online treatment for GAD symptoms be effective for an adult student population in a service-based setting? Secondly, what do participants find helpful and hindering in their online treatment for GAD symptoms? Thirdly, are participants satisfied with accessing and using an online treatment? Fourth and finally, what are the reasons for dropout from an online treatment?

Based on the success that has been achieved with supported online treatments in general [42,54,63,64] and with internet-delivered interventions for GAD [33,48-51,65], we hypothesize that participants in the trial will demonstrate significant decreases in GAD symptoms post-intervention and a corresponding positive change in pathological worry and quality of life.

## Methods/Design

### Study design

The research is a service-based effectiveness study utilizing a randomized controlled trial design of an internet intervention for the treatment of GAD symptoms. Participants will be randomized into two groups: the internet-delivered intervention with clinical support and a waiting list control group. The study protocol, information on the study, informed consent and related materials received ethical approval from the School of Psychology, Trinity College Dublin (22 November 2013).

### Sample size

Previous studies of online interventions for GAD that have used a CBT protocol similar to the intervention in the current study have reported post-treatment effect sizes of between 0.79 and 1.67, and follow-up effects of between 0.69 and 1.65, based on sample sizes ranging from 10 to 97 [33,48-50]. We calculated power using G-Power software [66]. Using a power of 0.80 and an alpha of 0.05, we would need 50 subjects in each arm of the trial to observe a moderate (*d* = 0.50) post-treatment difference on the main outcomes.

### Eligibility criteria

All registered students at the University of Dublin, Trinity College, Dublin, will be eligible to participate. The study will therefore consist of adult primary care patients fulfilling the Diagnostic and Statistical manual for Mental Health Disorders – Version 5 (DSM-5) [2] criteria for generalized anxiety symptoms. It is the case that all participants will have clinically meaningful generalized anxiety symptoms. Participants with comorbid disorders, such as mood disorders will be included once GAD is the primary diagnosis. On screening participants, eligibility criteria include that participants are at least 18 years of age and have a DSM-IV congruent score of 10 or above on GAD-7. Participants attending face-to-face counseling will be excluded.

### Recruitment

Registered students at the University of Dublin, Trinity College, Dublin, will receive an email advertising the study and inviting students to take part. Interested students will be able to visit a website to receive more information on the study, what will be involved in participating, the treatment, and how to make contact and proceed with screening. On agreeing to participate, informed consent will be completed online and thereafter the baseline screening questionnaires.

### Randomization

Using computer algorithms to score screening instruments at baseline, participants will be automatically randomized to either the active intervention group or a waiting list control group. Participants will be immediately informed about the randomization outcome. The randomization procedure will be managed by a person independent of the research group.

### Interventions

#### Silver Cloud platform

Delivered through the SilverCloud platform, the program for the treatment of GAD employs several innovative engagement strategies for improving the user experience. These are divided into several categories: personal, interactive, supportive, and social.

#### Personal

The user has his or her own secure homepage, and can fill in a profile with basic information about themselves. As well as establishing a sense of ownership, this information is also useful for the supporter, allowing the supporter to provide more personal feedback. The homepage is intended to provide a reflective space; the user can document their thoughts and feelings, and these can be elaborated on within the journal application, which also acts as the vehicle for therapeutic writing exercises. The user has actions suggested to them, and as they complete modules of the program their achievements are noted. Users are free to access the modules in any order they wish, in either a linear or non-linear manner, contributing to a sense of empowerment. Alongside the central content, a range of satellite applications are provided, such as a goal-setting application which can be used independently of the program content. Applications are released as the user completes modules, with the intention of maintaining engagement by introducing new features over time and not overwhelming the user initially. Users can also control which applications appear on their home page.

#### Interactive

The program includes a number of interactive elements and graphical exercises which are aimed at engaging users with the therapeutic content, for example, reflecting on their own thinking style. Users also have the ability to respond to content, indicating whether they like it, and also to comment on it. Both exercises and comments can be explicitly shared with the supporter. The user is provided with immediate feedback wherever possible; for example, when a charting exercise such as a mood chart is completed, the application item is graphically updated on the home page. Likewise, items are ticked off on the to-do list when completed and achievements are unlocked in each module summary.

#### Supportive

Each user has an assigned supporter who provides weekly reviews of their progress on the program. This support is asynchronous, whereby the supporter sets a date to review their user’s progress, and they do not provide feedback, support or contact outside this time. The supporter can support multiple users, logging in once weekly for instance, and reviewing the work of all their online users within an allocated time period. Such asynchronous online contact may be logistically easier to implement for many services compared to motivational interviewing and telephone support. The system supports the exchange of messages between the user and supporter, but goes beyond email as the user is encouraged to share their content (such as completed exercises and comments) with their supporter. This shared content allows the supporter to respond in a more personal way and provide guidance as well as encouragement to keep using the program. Adherence information is also available to the supporter, and they can keep track of the user’s progress. This is all personally sensitive information, and so a shared view is provided in the user interface where they can see the supporter’s view of their data. By making the visibility of user data to the supporter more transparent, as well as the ability to explicitly change the sharing status of data, the user is provided with a greater sense of control while facilitating a meaningful interaction with the supporter.

#### Social

While group therapy and peer group support are well established, introducing contact with other users within any online system raises a number of ethical concerns regarding the possibility for unhelpful or negative content or communications. As a first step, the user can see anonymous indications of other people in the system. The intention is to reassure users that they are not alone in experiencing difficulties and that many other people have experienced similar problems and overcome them. Users can respond to content by indicating that they ‘like’ it, and can see how many other people liked it, helping to reduce the sense of isolation. Other more detailed shared content (such as tips and ideas) is subject to supporter moderation.

### Computerized cognitive-behavior therapy (cCBT) program

*Calming Anxiety* is a six-module online CBT-based intervention for GAD. The structure and content of the program modules follow evidence-based principles of CBT for GAD treatment based on the AMW model of GAD [27]. The treatment comprises cognitive, emotional, and behavioral components that include self-monitoring, relaxation training, self-control desensitization, gradual stimulus control, cognitive restructuring, and worry outcome monitoring [26]. The treatment is delivered on a Web 2.0 platform using media-rich interactive content. The content of each module is described briefly in Table 1 below. Each module is structured in an identical way and incorporates introductory quizzes, videos, informational content, interactive activities, as well as homework suggestions and summaries. In addition, personal stories and accounts from other clients are incorporated into the presentation of the material.

**Table 1:** Calming anxiety: description of module content

**Module name**	**Brief description**
Getting Started	Outlines the basic premise of CBT and provides some information about anxiety. Users are encouraged to explore their current difficulties with anxiety and to begin monitoring their anxiety levels.
Understanding Moods and Emotions	This module describes the behavioral, physical, and emotional aspects of the Thoughts-Feelings-Behaviour (TFB) cycle. The user is introduced to relaxation practices. Users start to build their own anxiety-related TFB cycles
Anxious thoughts and worry	This module focuses on noticing anxious thoughts and worry, and ways of relating to these thoughts, including acceptance, distraction, and ‘worry time’.
Face your Anxiety, Step by Step	This module outlines why avoidance is harmful, and breaks down the steps needed for successful graded exposure. Users are encouraged to build their own fear hierarchies and to begin working through them.
Challenge your Anxious Thoughts	This module explains negative automatic thoughts, their role in anxiety, and how to challenge them. Users are encouraged to challenge the thoughts in their TFB cycles, and make use of helpful thoughts.
Bringing it all Together.	In this final module, users are encouraged to bring together all the skills and ideas they have gathered so far, note their personal warning signs, and make a plan for staying well.

### Waiting list control

**Figure 1:**
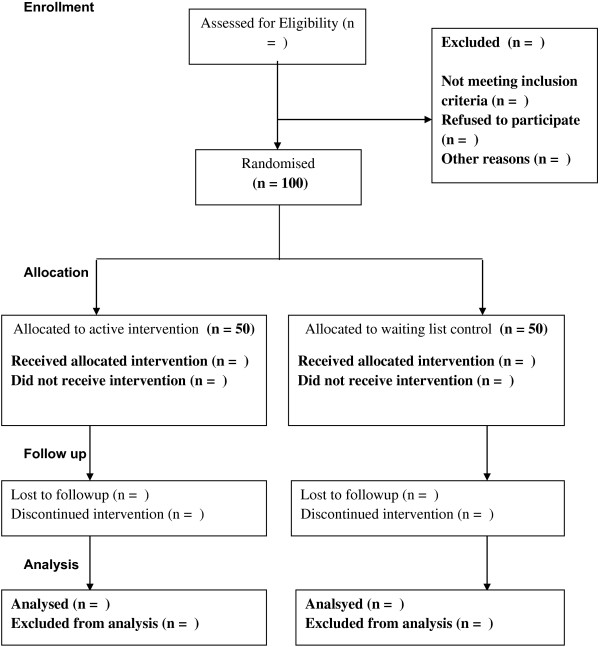
Flowchart of the study CONSORT.

Participants in the waiting list control group will not receive any treatment for the duration of the intervention for the immediate treatment group (six weeks). At week seven the waiting list participants will be given access to treatment under the same conditions as the immediate treatment group received (Figure 1).

### Support during treatment

Each participant will be assigned a supporter who will monitor participant’s progress throughout the trial. Once a participant is assigned to the active treatment condition at their first login there will be a message from their supporter. This message welcomes them to the program, highlights aspects of the program, and encourages them in the use of the program. Each week the supporters will login and review participants progress, leaving feedback for them and responding to the work they have completed. Participating supporters will receive training in the program and how to deliver feedback.

Supporters will be graduate psychology students and will be qualified to at least masters level, some with doctorates. Each supporter will be assigned participants to provide post-session feedback of between 10 and 15 minutes per participant per session.

### Assessments

At baseline, assessments (see Table 2) including Sociodemographics & History Questionnaire, Generalized Anxiety Disorder-7 (GAD-7), Penn State Worry Questionnaire (PSWQ), Beck Depression Inventory (BDI-II), Work and Social Adjustment Scale (WSAS), and EuroQol (ED-Q5) will be completed for screening purposes. Thereafter the GAD-7, PSWQ, BDI-II, ED-Q5 and WSAS will be completed at the end of treatment, week six (for both the active treatment group and the waiting list participants). The active treatment group will complete GAD-7, PSWQ, BDI-II, ED-Q5 and WSAS at the first follow-up, week 12 (three months). After each session supporters will provide post-session feedback. Thereafter participants will be asked to complete the Helpful Aspects of Therapy Form (HAT). The measure Satisfaction with Treatment (SAT) will be administered at week six.

**Table 2:** Study measures to be used

**Measure**	**Assessment**	**Time of assessment**
Generalized Anxiety Disorder-7 (GAD-7)	Anxiety symptoms	Baseline, post-treatment and follow-up
Sociodemographic & History Questionnaire	Gender, age, marital status, education, occupation, socioeconomic status, and history	Baseline
Penn State Worry Questionnaire (PSWQ)	Symptoms of anxious worry	Baseline, Post-treatment and follow-up
Beck Depression Inventory (BDI-II)	Depression symptoms	Baseline, post-treatment and follow-up
EuroQol (EQ-5D-5 L)	Quality of Life indicators	Baseline, post-treatment and follow-up
Work and Social Adjustment Scale (WSAS)	Work and Social Adjustment scale	Baseline, post-treatment and follow-up
Engagement and Usage data	Engagement and usage	Continuous
Satisfaction with Treatment (SAT)	Satisfaction with therapy	Post-treatment
Helpful and Hindering Aspects of Therapy (HAT)	Helpful and hindering aspects of therapy	After each session

### Measures

#### Primary outcome

The General Anxiety Disorder questionnaire (GAD-7) [67] comprises seven items measuring the symptoms and severity of GAD based on the DSM-IV diagnostic criteria for GAD. The GAD-7 has good internal consistency (0.89) and good convergent validity with other anxiety scales [68]. Increasing scores indicate a greater severity of symptoms [69]. Scores of 5, 10, and 15 are taken as the cutoff points for mild, moderate, and severe anxiety, respectively. The GAD-7 is increasingly used in large-scale studies as a generic measure of changes in anxiety symptomatology [37,70]. Using the threshold score of 10, the GAD-7 has a sensitivity of 89% and a specificity of 82% for GAD; it is considered congruent of DSM-IV as a clinical case for GAD [67].

#### Secondary outcomes

The Sociodemographic Information & History questionnaire is based on the History Questionnaire used in an earlier study [61]. It will be developed for the present study and will collect demographic details of the participants. It will collect data on any previous diagnosis of anxiety disorders and on the length of time that one experiences anxiety symptoms. It will collect data on participant’s experience of counseling and therapy, and medication for anxiety. The questionnaire will collect data on whether one has a previous diagnosis of an organic mental health disorder such as schizophrenia, psychosis, or bipolar disorder. In addition it contains items related to the comorbidity of anxiety with the presence of psychosis, alcohol and drug misuse, and/or any recent medical diagnosis.

The Penn State Worry Questionnaire (PSWQ) [66] consists of 16 items and is considered a valid clinical measure of the worry characteristic of GAD. Each item is measured on a 5-point Likert scale (1 - not at all typical of me to 5 - very typical of me) and a total score ranging between 0 and 80 is calculated by summing all items. Psychometric evaluations have revealed a high internal consistency (α = 0.86 to 0.95) and test-retest reliability over four weeks (*r* = 0.74 to 0.93) [71]. The measure is able to differentiate between patients with GAD and those with other anxiety disorders [72].

The 21-item Beck Depression Inventory - Second Edition (BDI-II) [73] is a widely used questionnaire developed for the assessment of depressive symptoms that correspond to the criteria for depressive disorder diagnosis as outlined in The American Psychiatric Associations Diagnostic and Statistical Manual of Mental Disorders - Fourth Edition (DSM-IV) [74]. Each item is scored on a scale from 0 to 3. The BDI-II manual states that a cutoff score of 17 has yielded a 93% specificity and 18% sensitivity for the presence of major depression (Beck *et al.*[73]). The scale designates levels of severity: minimal (0 to 13); mild (14–19); moderate (20 to 28); and severe (29 to 63) [73].

The BDI-II has been found to have an excellent internal consistency and test–retest reliability with a diverse range of samples [73,75,76]. The BDI-II has demonstrated a good convergent validity with other measures of depression among clinical and nonclinical adult samples [77].

The EuroQol 5D (EQ-5D) questionnaire [78] is a generic instrument of health-related quality of life. Part one records self-reported problems in each of five domains: mobility, self-care, usual activities, pain and/or discomfort and anxiety and/or depression. Each domain is divided into three levels of severity corresponding to no problems, some problems, and extreme problems, which allows a population-based preference score or societal index (SI) to be obtained. Part two records the subjects self-assessed health on a Visual Analogue Scale (VAS), a 10 cm vertical line on which the best and worst imaginable health states score 100 and 0, respectively.

The Work and Social Adjustment (WSA) questionnaire [79] is a simple, reliable, and valid measure of impaired functioning. It is a five-item self-report measure that provides an experiential impact of a disorder from the patient’s point of view. It looks at how the disorder impairs the patient’s ability to function day-to-day on five dimensions: work, social life, home life, private life, and close relationships.

### Other participant measures

The Helpful Aspects of Therapy Form (HAT) [80,81] is an instrument that assesses the most significant events in the therapy. Participants are asked to describe in their own words anything they engaged with in the session that was helpful or hindering for them.

The Satisfaction with Treatment (SAT) questionnaire [82] establishes a net promoter score that serves as a tool to gauge customer satisfaction. It includes one question: How likely is it that you would recommend this treatment to a friend or colleague? The measure also asks several other quantitative questions regarding satisfaction with accessing treatment online. The satisfaction measure contains two qualitative questions asking participants to describe what they most liked and least liked about the online treatment.

The pre-treatment and during treatment dropout questionnaires are two simple questionnaires, one asking about the reasons for deciding to dropout without beginning treatment and the second asking about the reasons for dropping out during treatment. The link for each of these will be contained in the appropriate emails that supporters send to their respective participants, following the protocol. Pre-treatment dropout: after one week the supporter can send the questionnaire by email. Participants discontinue treatment: after one missed session the supporter should send a reminder message to the participant by email. If after one further week the participant has not responded, the supporter can send the questionnaire asking about the reasons for dropout.

### Engagement and usage data

The online system will collect anonymized descriptive information relating to engagement and usage. Data collected will include the number of sessions completed, mean time spent on the program, average number of sessions per user, and average length of a session. A session is defined as an instance where a user logs onto the system. Session time estimation will always be an imperfect calculation, as users may be interrupted or take breaks within a session, and may not formally log out of the system. All client activity within the system such as reading content pages, saving a journal entry, or updating an activity, is logged with a time stamp. Starting with the log entry of the client logging on, the total time is calculated by adding up the time that elapses between each subsequent log record (in the same manner as popular web analytics software). On its own, this will yield a result vulnerable to overestimation of session time. To avoid counting periods where the user is not actively engaged with the system, any interactions taking longer than 30 minutes are counted as 1 minute. Any period of inactivity longer than three hours will start the count on a new session, rather than extending the time of the current session. Use of different program components will be measured. Data related to supporter reviews will be collected.

### Ethical considerations

Information made available to all prospective participants will inform them of exactly what is involved in participating, including the objectives of the trial and its importance. Participants will be informed of the importance of the waiting list control group. Informed consent will be obtained from each participant before randomization. Participants will know that their involvement is voluntary and they can withdraw their participation at any time without prejudice. Informed consent is collected online through participants digital signature and is returned online.

### Planned statistical analysis

The analyses will be based on the intention-to-treat principle, including those who began treatment and provided follow-up data irrespective of treatment compliance. Missing data will be handled using Last Observation Carried Forward (LOCF). Effects will be tested at the 0.05 level. Analysis of variance (ANOVA)will be used to investigate any baseline demographic or clinical differences between the groups.

To test the main hypotheses, repeated measures ANOVA will be performed for the primary outcome measure for anxiety (GAD-7). Thereafter ANOVAs will be executed for the BDI-II, PSWQ, EQ-5D and WSAS. Contrasts will be conducted comparing changes from baseline to post-treatment for each group separately. Further analysis of baseline demographic variables and any relation to outcomes will be conducted. Effect sizes (Cohen’s d) will be calculated both within and between groups, based on the pooled standard deviation. For Cohen's d an effect size of 0.2 to 0.3 can be considered a small effect, around 0.5 a medium effect, and 0.8 upwards a large effect [83].

Analysis will be made to determine the proportion of participants who make a clinically meaningful change at the end of treatment and at follow-up. Pre-treatment, post-treatment, and follow-up GAD-7, BDI-II and PSWQ scores will be compared with clinical cutoffs to provide an indicator of remission. Remission is defined as the number of participants who initially scored at or above the established clinical cutoffs, and then at post-treatment and follow-up scored below the established clinical cutoffs: GAD-7 total score <10 [67]; BDI-II total score <14 [84], PSWQ <45 [85], and WSAS <10 [79]. In addition, we will calculate the relative risk of anxiety and depression by dividing the event rate (anxiety or depression) post-treatment by the event rate pre-treatment [70]. An estimation of recovery will be made by identifying the number of participants in each group who demonstrated a reduction of 50% of pre-treatment GAD-7, BDI-II and PSWQ scores [70]. In addition we will also calculate the number of reliably changed and recovered participants using Jacobson and Truax’s [86] criteria.

HAT data will be analyzed qualitatively following the descriptive and interpretative framework described by Elliott and Timulak [87]. Participants’ responses will be considered within domains of helpful and non-helpful events and impacts. Firstly, individual units of text that could stand meaningfully out of their context will be identified. Next each of these will be organized into domains of helpful events and helpful impacts. Similar events and impacts will be grouped into categories, which will be then finalized and suitably named and defined. The process is organic, involving constant reference to the source data [88].

SAT data [82] will firstly establish the net promoter score. Descriptive statistics will be used to report on other quantitative questions, and qualitative data will be analyzed following the descriptive and interpretative framework described by Elliott and Timulak [87]. Similarly, data from dropout pre-treatment and during treatment will be analyzed qualitatively.

## Discussion

This study seeks to evaluate the effectiveness of an internet-delivered treatment for GAD in a sample of students in Ireland. The study will be a contribution to the potential for a low-intensity internet-delivered program implemented in a service-based setting. The study will be a contribution to the already established work in online treatments for anxiety worldwide.

The primary outcome measure (GAD-7) that will assess anxiety symptoms is a well-established measure and has been used in previous trials involving internet-delivered and face-to-face treatments. The secondary outcome measures (BDI-II, PSWQ) will each give an insight into participant’s improvements, principally in pathological worry (a central construct in GAD) and any corresponding improvements in comorbid depressive symptoms. The WSAS and ED-Q5 will assess any improvements in quality of life indicators corresponding to improvements in anxiety symptoms for participants.

The other secondary outcome measures (HAT, SAT) that we have included in the study will contribute to what participants find satisfying with online treatments and further will detail what in-session events and their impacts participants report as being helpful or hindering in their online treatments [82,88].

It is also hoped that with the inclusion of pre-treatment and during treatment dropout questionnaires, the study can make a contribution to developing a better understanding of the reasons for dropout in online treatments.

The results may not be generalizable to the wider community in Ireland, but perhaps may give insight into the usefulness of low-intensity internet-delivered interventions within a stepped-care model in routine primary care. The internet-based intervention is interesting for adult students who are high users of the internet and related tools, who look for alternative healthcare [53] as a preference, and because the possibility of accessing traditional services is difficult, prohibitive due to waiting lists, costs, and personal stigma.

## Trial status

This trial began in January 2014. We are currently beginning a second round of recruitment.

## Abbreviations

AMW: Avoidance Model of Worry; BDI-II: Beck Depression Inventory-II; CBT: Cognitive Behavior Therapy; EQ-5D: EuroQol; GAD: Generalized Anxiety Disorder; HAT: Helpful Aspects of Therapy; IUM: Intolerance of Uncertainty Model; PD/A: Panic Disorder with/without Agoraphobia; PSWQ: Penn State Worry Questionnaire; SAD: Social Anxiety Disorder; SAT: Satisfaction with Therapy; SP: Specific Phobia; WSAS: Work and Social Adjustment Scale.

## Competing interests

GD and JS, declare a minority interest in the commercialization of the SilverCloud platform.

## Authors’ contributions

DR, LT, GD and JS are principal investigators for the project. DR, LT, GD, JS conceptualized the initial trial design. This was developed with the help of OM, AC, CR and CJ. DR wrote the manuscript, with significant contributions and revisions from LT, GD, JS. DR distributed the manuscript to the entire group for discussion and revision and finalized with all agreed on the submitted manuscript. All authors have read and approved the manuscript.

## Author’s information

DR, SilverCloud Health Ltd. Dublin, Ireland & School of Psychology, Trinity College Dublin. OM, CR, Student Counseling, University of Dublin, Trinity College, Dublin. LT, School of Psychology, University of Dublin, Trinity College, Dublin. GD, School of Computer Science and Statistics, University of Dublin, Trinity College, Dublin. JS, Mater Misericordiae University Hospital, Dublin, Ireland. AC, CJ, SilverCloud Health Ltd. Dublin, Ireland.
